# Phylogenetic analysis of HA and NA genes of influenza A viruses in immunosuppressed inpatients in Beijing during the 2018–2020 influenza seasons

**DOI:** 10.1186/s12985-023-02067-2

**Published:** 2023-05-26

**Authors:** Yafen Liu, Yue Wang, Yanxin Wang, Huan Mai, YuanYuan Chen, Yifan Zhang, Ying Ji, Xu Cong, Yan Gao

**Affiliations:** 1 Department of Infectious Diseases, Peking University Hepatology Institute, Peking University People’s Hospital, No. 11, Xizhimen South Street, Xicheng District, Beijing, 100044 People’s Republic of China; 2 Peking University Hepatology Institute, Peking University People’s Hospital, No. 11, Xizhimen South Street, Xicheng District, Beijing, 100044 People’s Republic of China

**Keywords:** Influenza, Influenza A virus, Immunosuppression, Evolution, Variation

## Abstract

**Background:**

Influenza A viruses have undergone rapid evolution with virulent; however, complete and comprehensive data on gene evolution and amino acid variation of HA and NA in immunosuppressed patients was few. In this study, we analysed molecular epidemiology and evolution of influenza A viruses in immunosuppressed population, and immunocompetent population were used as controls.

**Methods:**

Full sequences of HA and NA of A(H1N1)pdm09 and A(H3N2) were acquired through reverse transcription-polymerase chain reaction (RT-PCR). HA and NA genes were sequenced using the Sanger method and phylogenetically analysed using ClustalW 2.10 and MEGA software version 11.0.

**Results:**

During the 2018–2020 influenza seasons, 54 immunosuppressed and 46 immunocompetent inpatients screened positive for influenza A viruses by using the quantitative real-time PCR (qRT-PCR) were enrolled. 27 immunosuppressed and 23 immunocompetent nasal swab or bronchoalveolar lavage fluid samples were randomly selected and sequenced using the Sanger method. A(H1N1)pdm09 were detected in 15 samples and the remaining 35 samples were A(H3N2) positive. By analyzing the HA and NA gene sequences of these virus strains, we found that all A(H1N1)pdm09 viruses shared high similarities to each other and the HA and NA genes of these viruses exclusively belonged to subclade 6B.1A.1. Some NA genes of A(H3N2) viruses were not in the same clade as those of A/Singapore/INFIMH-16-0019/2016 and A/Kansas/14/2017, which may have led to A(H3N2) being the dominant strain in the 2019–2020 influenza season. Both A(H1N1)pdm09 and A(H3N2) viruses showed similar evolutionary lineages patterns of HA and NA between immunosuppressed and immunocompetent patients. Compared with the vaccine strains, there were no statistically significant of HA and NA genes and amino acid sequences of influenza A viruses in immunosuppressed and immunocompetent patients. However, the oseltamivir resistance substitution of NA-H275Y and R292K have been observed in immunosuppressed patients.

**Conclusions:**

A(H1N1)pdm09 and A(H3N2) viruses showed similar evolutionary lineages patterns of HA and NA between immunosuppressed and immunocompetent patients. Both immunocompetent and immunosuppressed patients have some key substitutions, which should be of note monitored, especially those with potential to affect the viral antigen.

**Supplementary Information:**

The online version contains supplementary material available at 10.1186/s12985-023-02067-2.

## Background

Influenza viruses include A, B, C and D, and among these four types, type A is the most infectious, which can even cause a life-threatening pandemic [1]. A (H1N1) pdm09 and H3N2 are currently main circulating strains of influenza A virus. The hemagglutinin (HA) gene has the fastest mutation of the eight genes in influenza A viruses, followed by the neuraminidase (NA) gene [2]. The main mechanism of influenza A virus variation are antigenic drift and genetic reassortments [3]. Previous studies have shown that influenza A(H1N1)pdm09 was derived from several swine influenza viruses and transmitted to humans in the months prior to the outbreak [4, 5]. Active monitoring of influenza virus at the molecular level can help to understand the evolution of influenza virus and select better vaccine strains [6, 7].

The previous literatures and surveillances of the Centers for Disease Control and Prevention (CDC) of influenza at the molecular level were mainly from immunocompetent patients [8, 9]. With the progress of medical technology, immunosuppressed patients who have undergone haemopoietic stem cell transplantation (HSCT) or solid-organ transplantation (SOT), patients on chronic haemodialysis, and patients receiving systemic corticosteroids, immunosuppressants and biological reagents have increased each year [10–12]. Immunosuppressed patients accounted for 10.3% of the global multi-center cross-sectional study of 35,348 adult influenza patients published in 2020 [13]. Complete and comprehensive data on influenza gene evolution and amino acid variation of HA and NA in immunosuppressed patients are lacking, and it is unclear whether they differ from patients with immunocompetent patients.

Variations in HA and NA may lead to the emergence of new clinical features, such as the HA-D239 site mutation is associated with severity of illness, while NA-H275, E119, R292, Q136, and I223 substitutions can lead to neuraminidase inhibitors (NAIs) resistance [14–16]. Compared with immunocompetent patients, immunosuppressed patients have higher morbidity and mortality, a longer duration of viral shedding, more frequent complications, and more antiviral resistance [10, 17–21]. It is necessary to comprehensively monitor gene evolution and amino acid variation of HA and NA in immunosuppressed patients, and explore whether it has an effect on clinical characteristics and outcome of these patients.

Clinical characteristics and antiviral therapy for immunosuppressed influenza patients were analysed in our prior study [22], and in this study, we further investigate the molecular evolution and amino acid variation of HA and NA of influenza A viruses during the 2018–2020 influenza seasons in these population. In addition, we intended to explore vital amino acid variations of HA and NA whether have an effect on patients’ clinical characteristics and outcomes.

## Materials and methods

### Patients and sample collection

Peking University People’s Hospital (PKUPH) is a national influenza surveillance sentinel unit, receiving at least 100,000 inpatients from all Beijing districts annually. During the 2018–2020 influenza seasons (November to the following March), 54 immunosuppressed and 46 immunocompetent inpatients screened positive for influenza A viruses by using the quantitative real-time PCR (qRT-PCR) method were enrolled in this study. Original nasal swab or bronchoalveolar lavage fluid (BALF) samples from these patients were collected and immediately placed in virus transport media tubes for analysis. Immunosuppressed patients were defined as the presence at least one risk factor as follows: congenital/genetic immunocompromise, HIV infection, SOT, HSCT, malignancies receiving chemotherapy, aplastic anemia, chronic haemodialysis, chronic steroid use, immunosuppressive agents use and biological drug use [10–12]. Full sequences of HA and NA of A(H1N1)pdm09 and A(H3N2) were acquired through reverse transcription-polymerase chain reaction (RT–PCR) method [23].

### Data collection

Data on demographic factors (sex, age, cause of immunosuppression, diabetes, use of corticosteroids in the preceding 3 months, neuraminidase inhibitor use before admission), clinical presentation and complications (maximum body temperature, headache, muscle soreness, rhinorrhoea, sore throat, cough, dyspnoea, gastrointestinal symptoms, altered mental status, symptom onset, coinfection with other pathogens, complications, antiviral treatment, admission to the intensive care unit (ICU), mechanical ventilation, time to fever clearance, death) and laboratory test results [white blood cell (WBC), lymphocyte, and C-reactive protein] were collected through medical records.

### RNA extraction and reverse transcription

We extracted RNA from samples using the QIAamp Viral RNA Mini Kit (Cat. No.52904, Qiagen, Hilden, Germany) and performed the reverse transcription with a commercial kit (Cat. No.18080051, Invitrogen, Carlsbad, CA, USA), following the manufacturer’s instructions. The complementary DNAs (cDNAs) generated from the reverse transcription were stored at − 20 °C until use.

### Gene sequencing of HA and NA

For sequencing the HA and NA genes, high-fidelity thermostable DNA polymerase (Cat. No.11304011, Invitrogen) was used. Specific primers for HA and NA were shown in Table 1. The PCR amplification system included the cDNA template (4 μl), Autoclaved, distilled water (12.1 μl), 10X High Fidelity PCR Buffer (2 μl), 50 mM MgSO_4_ (0.6 μl), 10 mM dNTP Mix (0.4 μl), 10 µM forward primer (0.4 μl), 10 µM reverse primer (0.4 μl), and Platinum® Taq DNA Polymerase High Fidelity (0.1 μl of 5U/µL). The PCR conditions were: 94 °C for 3 min, followed by 40 cycles of 94 °C for 15 s, 60 °C for 30 s and 72 °C for 2 min, with extension at 72 °C for 10 min. PCR products were analyzed by the method of electrophoresis. 27 immunosuppressed and 23 immunocompetent samples were randomly selected and PCR products were directly sequenced using the Sanger method (BioGerm, Shanghai).

**Table 1:** PCR primers for HA and NA full genome sequencing of A(H1N1) pdm09 and A(H3N2)

Primer name	Primer sequence (5’–3’)
A(H1N1) pdm09	
pdmHA-I-F4	TGTAAAACGACGGCCAGTAAAAGCAGGGGAAAACAAAAGC
pdmHA-I-R1049	CAGGAAACAGCTATGACCYGGGRCATTCCTCAATCCT
pdmHA-II-F936	TGTAAAACGACGGCCAGTGCTATAAACACCAGCCTCCC
pdmHA-II-R1756	CAGGAAACAGCTATGACCCTCATGMTTCTGAAATCCT
pdmNA-I-F9	TGTAAAACGACGGCCAGTCAGGAGTTTAAAATG
pdmNA-I-R869	CAGGAAACAGCTATGACCAGGRTARCAGGAGCATTCCTC
pdmNA-II-F741	TGTAAAACGACGGCCAGTRTAATGACYGAYGG
pdmNA-II-R1457	CAGGAAACAGCTATGACCAGTAGAAACAAGGAGTTTTT
A(H3N2)	
H3-I-F2	TGTAAAACGACGGCCAGTGCAAAAGCAGGGGATAATTC
H3-I-R964	CAGGAAACAGCTATGACCTTTTGRAATGGTTTGTCATTGG
H3-II-F868	TGTAAAACGACGGCCAGTAAGCTCRATAATGAGRTCAGAT
H3-II-R1788	CAGGAAACAGCTATGACCAGTAGAAACAAGGGTGTTTT
N2-I-F1	TGTAAAACGACGGCCAGTAGCARAAGCAGGAGT
N2-I-R982	CAGGAAACAGCTATGACCAAGTCCTGAACACACATAAC
N2-II-F879	TGTAAAACGACGGCCAGTTCAGATGTRTHTGCMGAGAC
N2-II-R1465	CAGGAAACAGCTATGACCAGTAGAAACAAGGAGTTTTT

### Phylogenetic analysis of HA and NA genes

The nucleotide sequences generated were aligned with publicly available sequences of influenza A viruses available in the National Center for Biotechnology Information (NCBI) and Global Initiative of Sharing All Influenza Data (GISAID) database. Full-length reference sequences, which were phylogenetically related to the H1N1 and H3N2 viruses, were included with our sequences for phylogenic analysis using MEGA v.11.0. Sequences with (a) evidence of lab errors and (b) 100% similarity were discarded. All HA and NA gene sequences were aligned using ClustalW 2.10. Phylogenetic analysis of HA and NA gene sequences and substitution analysis of HA and NA proteins were performed using MEGA software version 11.0. Sequences of HA and NA genes in our study have been deposited into NCBI with the accession number OQ455958-455972 and OQ456032-456116.

### Statistical analysis

Statistical analysis was performed using SPSS statistical software version 22.0 (SPSS Inc., Chicago, IL, USA). Two-group comparisons of normally distributed data were performed with the independent samples t-test. Frequency comparisons were made with the χ^2^ test. *P* values < 0.05 were considered statistically significant.

## Results

### Epidemics characteristics of influenza virus

**Fig. 1:**
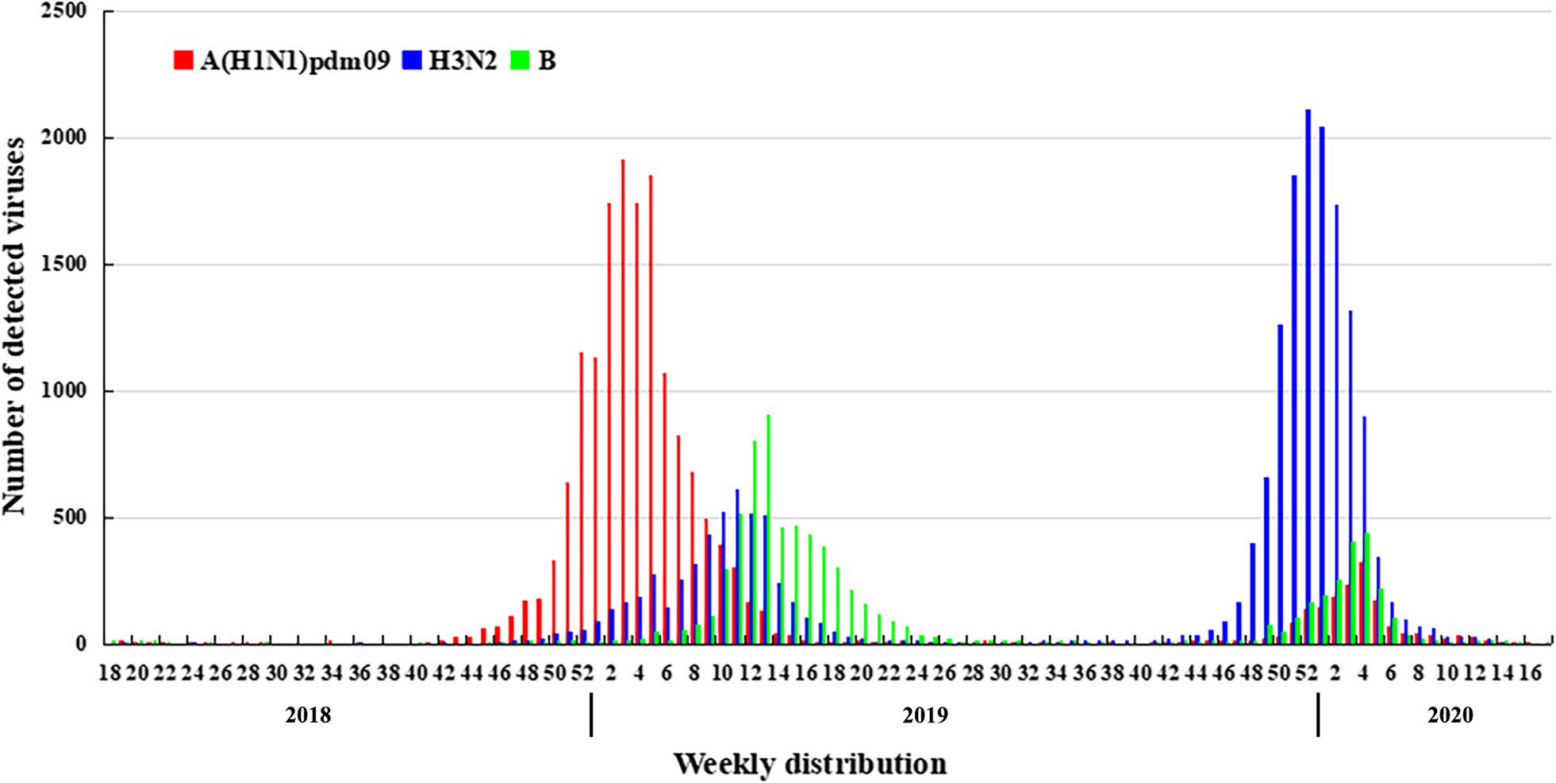
Distribution of influenza viruses in northern China. The data were from weekly data of Influenza Laboratory Surveillance Network during April 2018 to May 2020. Red is A(H1N1)pdm09, green is influenza H3N2, and blue is influenza B

According to the weekly influenza data released by Chinese National Influenza Center, the epidemiology of influenza virus in northern China from April 2018 to May 2020 is different in the 2018–2019 and 2019–2020 influenza seasons. As shown in Fig. 1, in the 2018–2019 influenza season, the epidemic of A(H1N1)pdm09 started early, peaked at the end of January, and began to decline in February, while A(H3N2) and B gradually increased, and both A(H3N2) and B showed small peaks at the end of March. In the 2019–2020 influenza season, A(H3N2) was dominant and peaked in early January. In the same period, although A(H1N1)pdm09 and B influenza viruses co-existed, they were significantly less than A(H3N2). In February 2020, control measures were taken against the coronavirus disease outbreak, and the circulation of many respiratory diseases was significantly reduced including influenza [24–27].

### Distribution of immunosuppressed factors

During the 2018–2020 influenza seasons, 54 immunosuppressed and 46 immunocompetent inpatients were verified using RT–PCR. Our hospital was not designated hospitals of HIV infection, so patients with HIV infection were not involved in this study. The immunosuppressed factors in this study were as follows: 38 patients with malignancies receiving chemotherapy, 8 patients with chronic steroid use (4 patients also have a history of immunosuppressive or biological agents), 6 patients with HSCT (including 1 patient with aplastic anemia), and 2 patients with chronic haemodialysis. There was no congenital/genetic immunocompromise.

### Phylogenetic analysis of HA and NA genes

Twenty-seven inpatients with immunosuppression and 23 immunocompetent inpatients of our study were randomly selected and sequenced using the Sanger method. A(H1N1)pdm09 were detected in 15 samples and the remaining 35 samples were A(H3N2) positive. HA and NA gene sequences were compared with other A(H1N1)pdm09 and A(H3N2) sequences on NCBI and GISAID.

**Fig. 2:**
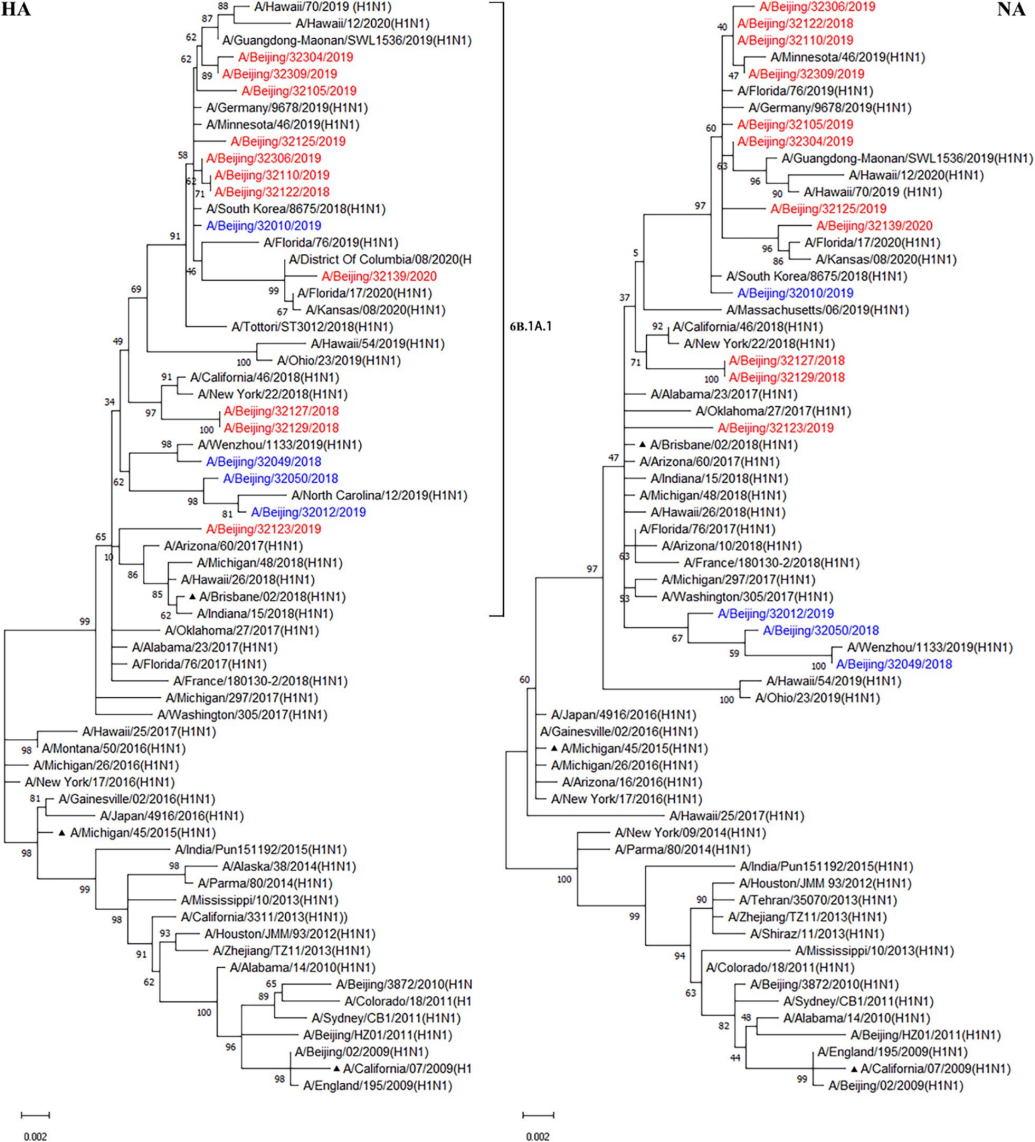
Phylogenetic tree based on HA and NA nucleotide sequences of A(H1N1)pdm09 from 2009 to 2020. Phylogenetic analysis of HA and NA gene sequences was performed with the Hasegawa-Kishino-Yano and Tamura 3-parameter model respectively which were the best fit for our data using MEGA software version 11.0, with gamma-distributed rates. The reliability of the maximum-likelihood tree was run by bootstrap analysis with 1000 replications. Filled triangle represents the vaccine strain. Red represents viral strains from immunosuppressed patients and blue represents viral strains from immunocompetent patients

Phylogenetic analyses of the HA genes showed the tested A(H1N1)pdm09 viruses felled together with the vaccine strain A/Brisbane/02/2018 rather than A/Michigan/45/2015 (H1N1), and belonged to subclade 6B.1A.1 (Fig. 2). Like HA genes, the NA genes showed the same evolutionary pattern. These results suggest that these viruses shared common evolutionary lineages to the vaccine strain A/Brisbane/02/2018, and HA and NA of A(H1N1)pdm09 viruses showed similar evolutionary lineages patterns between immunosuppressed and immunocompetent patients.

**Fig. 3:**
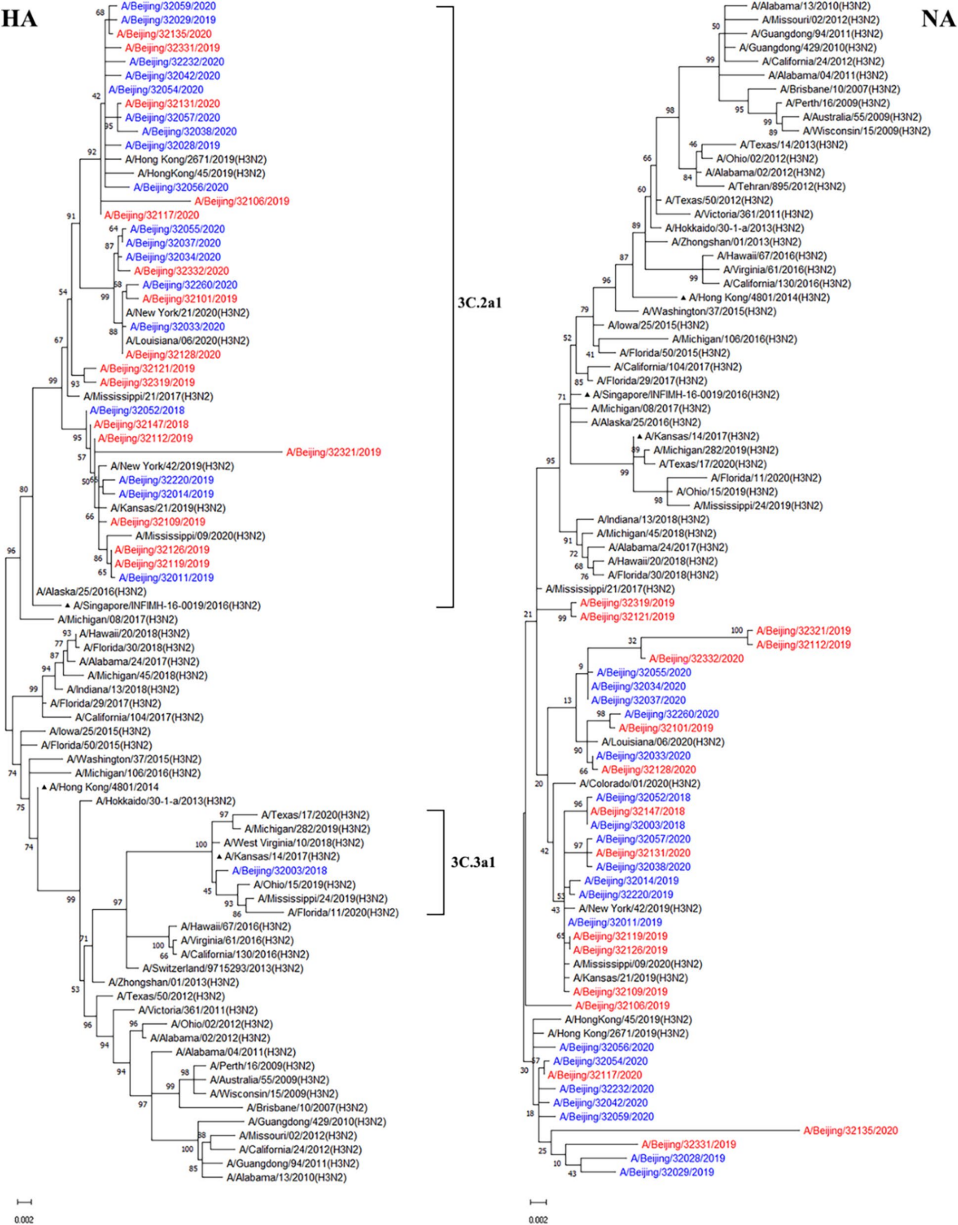
Phylogenetic tree based on HA and NA nucleotide sequences of A(H3N2) from 2009 to 2020. Phylogenetic analysis of HA and NA gene sequences was performed with the General Time Reversible and Tamura 3-parameter model respectively which were the best fit for our data using MEGA software version 11.0, with gamma-distributed rates. The reliability of the maximum-likelihood tree was run by bootstrap analysis with 1000 replications. Filled triangle represents the vaccine strain. Red represents viral strains from immunosuppressed patients and blue represents viral strains from immunocompetent patients

Phylogenetic analyses of the NA genes showed the tested A(H3N2) viruses felled together with the vaccine strain A/Singapore/INFIMH-16-0019/2016 except A/Beijing/32003/2018 which felled into A/Kansas/14/2017 (Fig. 3). While some NA genes of A(H3N2) viruses were not in the same clade as those of A/Singapore/INFIMH-16–0019/2016 and A/Kansas/14/2017, which may have led to A(H3N2) being the dominant strain in the 2019–2020 influenza season. HA and NA of A(H3N2) viruses also showed similar evolutionary lineages patterns between immunosuppressed and immunocompetent patients.

### Homology analysis of HA and NA genes

The HA and NA genes of the 15 A(H1N1)pdm09 and 35 A(H3N2) viruses were fully sequenced and analysed of homology. HA genes of these A(H1N1)pdm09 viruses shared 98.00% to 100.00% nucleotide similarity and 97.53% to 100.00% amino acid identity to each other. NA genes of these A(H1N1)pdm09 viruses shared 98.47% to 100.00% nucleotide similarity and 96.81% to 100.00% amino acid identity to each other. As shown in Additional files 1 and 2 Supplementary Tables 1, 2, A(H1N1)pdm09 isolates in this study shared 97.41% to 98.94% nucleotide similarity and 97.23% to 99.12% amino acid identity of their HA and NA genes to A/Michigan/45/2015 (H1N1) vaccine strain, respectively. Moreover, A(H1N1)pdm09 isolates shared 98.24% to 99.36% nucleotide similarity and 97.53% to 99.36% amino acid identity of their HA and NA genes to A/Brisbane/02/2018 vaccine strain, respectively. These results suggest that HA and NA of these viruses contained similar gene constellation and possessed high gene identity to the vaccine strain A/Brisbane/02/2018.

**Table 2:** Key substitutions of A(H1N1)pdm09 virus in this study

HA	Number of substitutions		NA	Number of substitutions	
	immunosuppressed	immunocompetent		immunosuppressed	immunocompetent
S127L	0	2	I99V	1	0
T137A	2	0	N222K	1	0
N146D	9	1	V267A	0	2
K147N	1	0	H275Y	1	0
A158E	1	0	G298A	1	0
N173K	1	0	S340F	1	0
V190I	0	1	N341D	0	1
S202I	8	1	I389K	8	0
K226M	1	0	V394I	1	0
M274I	1	0	S400T	0	2
N277D	8	1	T452I	8	1
			I467V	0	2

HA genes of these A(H3N2) viruses shared 94.59% to 100.00% nucleotide similarity and 93.83% to 100.00% amino acid identity to each other. NA genes of these A(H3N2) viruses shared 95.82% to 100.00% nucleotide similarity and 95.11% to 100.00% amino acid identity to each other. As shown in Additional files 3 and 4 Supplementary Tables 3, 4, A(H3N2) isolates in this study shared 95.67% to 98.88% nucleotide similarity and 94.47% to 98.24% amino acid identity of their HA and NA genes to A/Singapore/INFIMH-16-0019/2016 vaccine strain, respectively. Moreover, A(H3N2) isolates shared 94.71% to 99.76% nucleotide similarity and 93.83% to 100.00% amino acid identity of their HA and NA genes to A/Kansas/14/2017 vaccine strain, respectively. These results suggest that HA and NA of these viruses contained similar gene sequences, but some of which were different from above vaccine strains.

**Table 3:** Key substitutions of A(H3N2) virus in this study

HA	Number of substitutions		NA	Number of substitutions	
	Immunosuppressed	Immunocompetent		Immunosuppressed	Immunocompetent
G94S	1	0	G93S	1	3
Q96H	0	1	N141S	0	2
S140N	0	1	I257V	0	2
I156L	0	1	V263I	5	6
I156K	1	0	E277K	2	0
V198I	1	0	V287G	0	1
Q213R	2	1	R292K	2	0
S214P	3	5	R394K	1	0
I230V	1	0	R400K	1	0
N312S	0	1	S450L	1	0

Further comparisons between these two groups, we found that there were no statistically significant of HA and NA genes and amino acid sequences of influenza A viruses in immunosuppressed and immunocompetent patients (Additional files 1, 2, 3 and 4 Supplementary Tables 1, 2, 3, 4).

### Substitution analysis of HA and NA proteins

Compared with the vaccine strains of A/California/07/2009, A/Michigan/45/2015 and A/Brisbane/02/2018, HA of A(H1N1)pdm09 virus in this study exhibited 11 key substitutions, and NA of A(H1N1)pdm09 virus exhibited 12 key substitutions, as shown in Table 2. Furthermore, the oseltamivir resistance substitution of NA-H275Y have been observed in one immunosuppressed patients.

Compared with the vaccine strains of A/Hong Kong/4801/2014, A/Singapore/INFIMH-16–0019/2016, and A/Kansas/14/2017, HA of A(H3N2) virus in this study exhibited 10 key substitutions, and NA of A(H3N2) virus also exhibited 10 key substitutions, as shown in Table 3. Two virus strains have been observed oseltamivir resistance substitution NA- R292K in immunosuppressed patients.

### Clinical characteristics of influenza A infections with key amino acid variations

Both immunocompetent and immunosuppressed patients have some key amino acid variations. The oseltamivir resistance substitution of NA-H275Y and NA-R292K have been observed in immunosuppressed patients. One A(H1N1)pdm09 virus strain showed V190I mutation at the HA receptor binding site 190 helix. HA-A158E mutation of A(H1N1)pdm09 and I156L/K mutation of A(H3N2) were also be found. V190I and I156L mutation came from immunocompetent patients, while A158E and I156K mutation came from immunosuppressed patients. N222K and G298A near enzyme active center of NA in A(H1N1)pdm09 were also derived from immunosuppressed patients.

In this study, we described clinical characteristics of influenza A infections with vital amino acid variations (Additional file 5: Supplementary Table 5). NA-H275Y of A(H1N1)pdm09 came from a HSCT patients with aplastic anemia, who had oseltamivir and hormone exposure before influenza detection, complicated with coinfections, pneumonia and ARDS, and admitted to intensive care unit (ICU). He was treated with double dose of oseltamivir combined with peramivir on the basis of antibiotics and hormone therapy, and body temperature returned to normal 10 days later. NA-R292K of A(H3N2) came from patients receiving chemotherapy for hematopoietic malignancies and breast cancer respectively. None of these two patients had a history of exposure to NAIs before influenza detection. The former had a peak temperature of 40℃, complicated with pneumonia and acute kidney injury, and was treated with standard dose of peramivir for 2 days and oseltamivir for 5 days, and body temperature returned to normal 5 days later. The latter had a peak temperature of 37.7℃, a history of diabetes and no influenza-related complications. She was treated with standard dose of oseltamivir for 5 days, and his fever cleared after 2 days of treatment. Co-occurring mutations of HA and NA occurred in some patients with different clinical characteristics, for example, patient with co-occurring mutations of HA-A158E, NA-I99V and NA-G298A in A(H1N1)pdm09 did not have influenza-related complications such as pneumonia, and fever cleared 3 days after treatment with standard dose of oseltamivir. However, patient with co-occurring mutations of HA-M274I and NA-S340F complicated with pneumonia and ARDS, and were admitted to ICU. Fever cleared 12 days after treatment with oseltamivir and peramivir. NA-N141S mutant strains of A(H3N2) were accompanied by NA-I257V mutation, and NA-R292K mutant strains were accompanied by NA-E277K mutation.

## Discussion

Influenza A virus had a significant impact on public health during the 2018–2020 influenza seasons (Fig. 1). The genetic evolution of influenza virus is gradual, and our team conducted active monitoring of influenza at the molecular level since 2012 [28–31]. In this study, we analysed molecular epidemiology and evolution of HA and NA of influenza A viruses in immunosuppressed population, and immunocompetent population were used as controls. Phylogenetic analyses of HA and NA genes showed the tested A(H1N1)pdm09 viruses fell together with the vaccine strain A/Brisbane/02/2018 rather than A/Michigan/45/2015 (H1N1) during 2018–2020, and belonged to subclade 6B.1A.1, which was basically consistent with foreign reports [32]. Some NA genes of A(H3N2) viruses were not in the same clade as those of A/Singapore/INFIMH-16–0019/2016 and A/Kansas/14/2017, which may have led to A(H3N2) being the dominant strain in the 2019–2020 influenza season. Both A(H1N1)pdm09 and A(H3N2) viruses showed similar evolutionary lineages patterns of HA and NA between immunosuppressed and immunocompetent patients. Compared with the vaccine strains, there were no statistically significant of HA and NA genes and amino acid sequences of influenza A viruses in immunosuppressed and immunocompetent patients. However, the oseltamivir resistance substitution of NA-H275Y and R292K have been observed in immunosuppressed patients.

The epitopes that affect the antigenicity of influenza are mostly located on the surface of HA protein. Antigenic epitopes of A(H1N1)pdm09 include Sa, Sb, Ca, Cb, Pa and Pb, and antigenic epitopes of A(H3N2) include A, B, C, D and E. Both A(H1N1)pdm09 and A(H3N2) have same receptor-binding domain (RBD) on HA, which is composed of 190 helices, 130 rings and 220 rings [33]. In this study, HA-N146D, N173K and K226M substitutions of A(H1N1)pdm09 were at the Ca epitope, A158E was at the Sa epitope, V190I was at Sb epitope, and M274I was at Pa epitope. HA-G94S substitutions of A(H3N2) were located at E epitopes, Q96H, Q213R, S214P and I230V were located at D epitopes, S140N was located at A epitopes, I156L, I156K and V198I were located at B epitopes, and N312S were located at C epitopes. Above mutations of these epitopes may affect the antigenic characteristics of the virus. It has been reported that sites 132, 133, 135, 189, 190, 192, 193, and 197 are aligned to form an antigenic ridge on RBD, which is directly involved in receptor ligand interaction. Other candidate sites (e.g. 155, 156, 158, and 159) are located on a ring surrounding RBD, which can play an important role in immunization [33]. In this study, one A(H1N1)pdm09 virus strain showed V190I mutation at the HA receptor binding site 190 helix. HA-A158E mutation of A(H1N1)pdm09 and I156L/K mutation of A(H3N2) were also be found. In addition to RBS, vestigial esterase domain (ED) also participated in antigenic drift [33], and the variation of G94S derived from a patient with leukemia were located in the ED domain. The 220-ring mutation is often associated with host specificity. For example, leucine at 226 in humans preferentially recognizes alpha-2,6 sialic acid receptors, and glutamine at 226 in birds preferentially recognizes alpha-2,3 sialic acid receptors [34]. In this study, we got H1-K226M mutation from a patient on long-term hormone therapy.

Activity of NA protein is an important component of influenza virus infection. The epitopes of NA protein consist of 83–143, 156–190, 252–303, 330, 332, 340–345, 368, 370, 387–395, 400, 431–435 and 448–468. The enzyme catalytic sites of NA protein include the central site of enzyme activity (R118, D151, R152, R225, E277, R293, R368, Y402) and the auxiliary site (E119, R156, W179, S180, D/N199, I223, E228, H275, E278, N295, E425) [35]. In this study, NA of A(H1N1)pdm09 virus exhibited 12 significant substitutions, and N222K and G298A are close to the enzyme active center. Among of these substitutions, I99V, G298A, S340F and N341D have not been reported in the past. NA-I99V and G298A appeared in one lung cancer chemotherapy patients with HA-A158E, and S340F appeared in one leukemia patients with HA-M274I. Patients with co-occurring mutations of HA-A158E, NA-I99V and NA-G298A in A(H1N1)pdm09 did not have influenza-related complications; however patients with co-occurring mutations of HA-M274I and NA-S340F complicated with pneumonia and ARDS, and were admitted to ICU. HA and NA have complementary and antagonistic effects. Both HA and SA binding affinity and NA enzyme activity may disrupt the balance between HA and NA, but the co-occurring mutations of HA and NA genes may compensate for such imbalance [34, 36–38], which requires further study.

One NA-H275Y mutation and two NA-R292K mutations were found, which were the first time that our team found drug-resistant mutations at H275 and R292 sites. In order for oseltamivir to bind correctly, NA must be rearranged to form a pocket, and the key of rearrangements is E277 rotation so as to bind to R225. Vitro modeling and X-ray crystallography have shown that H275Y inhibits the rotation of E277 residues, thereby preventing the formation of pockets [39]. In this study, it was found that the virus strain with R292K mutation was accompanied by E277K mutation. Whether the resistance mechanism is similar to that of H275Y needs further study. In addition to the influence of NAIs, NA subtypes of influenza viruses may also contribute to mutate. H275Y is dominant in N1 subtypes, such as A (H1N1) and A (H5N1), while R292K are molecular markers of oseltamivir resistance of A(H3N2) virus [40, 41]. Immunosuppressed influenza patients have a long virus shedding time, and long-term exposure to neuraminidase inhibitors can promote the selection of drug-resistant variants [42, 43]. In this study, NA-H275Y of A(H1N1)pdm09 came from a HSCT patients with aplastic anemia, who had oseltamivir exposure before influenza detection, complicated with coinfections, pneumonia and ARDS, and admitted to ICU. NA-R292K of A(H3N2) came from patients receiving chemotherapy for hematopoietic malignancies and breast cancer respectively. None of these two patients had a history of exposure to neuraminase inhibitors before influenza detection. Cases of oseltamivir-resistant A(H1N1)pdm09 initially appeared mainly in immunocompromised patients receiving oseltamivir treatment, later in the 2010–2011 influenza season in the UK and other parts of the world, an increasing number of oseltamivir-resistant patients were found to have no history of oseltamivir use [44]. Peramivir binds to sialic acid residues in a manner similar to oseltamivir and is also affected by H275Y mutations, so combination of two NAIs was not recommended [45]. Double dose application of NAIs was recommended for immunosuppressed patients in earlier studies [19], but current studies have found that patients with double dose of NAIs have good tolerance, but no virologic or clinical advantage were observed [46–48]. If symptoms persist and tests consistently positive for the virus, especially in immunosuppressed patients, a longer course of treatment may be considered [23]. Due to some differences in the structure of NA, influenza A(H3N2) is less likely to show NAIs resistance than influenza A(H1N1)pdm09, which generally does not lead to significant loss of NA enzymology function and decreased viral fitness [44]. The clinical outcome of patients with R292K mutation in this study was consistent with this.

## Conclusions

This study provided complete and comprehensive data on gene evolution and amino acid variation of HA and NA in immunosuppressed patients with influenza A infections, and described clinical characteristics of key amino acid variations of HA and NA. A(H1N1)pdm09 and A(H3N2) viruses showed similar evolutionary lineages patterns of HA and NA between immunosuppressed and immunocompetent patients. Both immunocompetent and immunosuppressed patients have some key substitutions, which should be of note monitored, especially those with potential to affect the viral antigen. However, the number of this study may not be sufficient to draw the firm conclusions, further studies with a larger sample size will be needed to confirm and extend our findings.

## Supplementary Information

**Additional file 1: Table 1**. Nucleotide similarity of HA and NA genes of Apdm09 compared to vaccine strains.

**Additional file 2: Table 2**. Amino acid similarity of HA and NA genes of Apdm09 compared to vaccine strains.

**Additional file 3: Table 3**. Nucleotide similarity of HA and NA genes of Acompared to vaccine strains.

**Additional file 4: Table 4**. Amino acid similarity of HA and NA genes of Acompared to vaccine strains

**Additional file 5: Table 5**. Clinical characteristics of influenza A infections with key amino acid variations.

## Data Availability

The datasets used and/or analysed in the current study are available from the corresponding author upon reasonable request.

## Abbreviations

CDC: Centers for Disease Control and Prevention
ED: Esterase domain
GISAID: Global Initiative of Sharing All Influenza Data
HA: Hemagglutinin
HSCT: Haemopoietic stem cell transplantation
ICU: Intensive care unit
NA: Neuraminidase
NAIs: Neuraminidase inhibitors
NCBI: National Center for Biotechnology Information
PKUPH: Peking University People’s Hospital
qRT-PCR: Quantitative real-time PCR
RBD: Receptor-binding domain
RT–PCR: Reverse transcriptase–polymerase chain reaction
SOT: Solid-organ transplantation
μl: Microlitre
WBC: White blood cell
