# Supplementary material for: Phylogenetic analysis of HA and NA genes of influenza A viruses in immunosuppressed inpatients in Beijing during the 2018–2020 influenza seasons
Source: Virol J. 2023 May 26;20:101. doi: 10.1186/s12985-023-02067-2 (PMC10215044; doi:10.1186/s12985-023-02067-2)
Supplement: Supplementary file 2 — Additional file 2: Table 2. Amino acid similarity of HA and NA genes of Apdm09 compared to vaccine strains. [file 12985_2023_2067_MOESM2_ESM.docx]

| **Supplementary Table 2** Amino acid similarity of HA and NA genes of A(H1N1)pdm09 compared to vaccine strains | | | |
| --- | --- | --- | --- |
|  |  | MI45 | BR02 |
| HA | All virus strains | 97.35%-99.12% | 97.53%-98.94% |
|  | Virus strains from immunosuppressed patients | 97.35%-99.12% | 97.53%-98.94% |
|  | Virus strains from immunocompetent patients | 98.59%-98.77% | 98.59%-98.94% |
| NA | All virus strains | 97.23%-98.94% | 98.09%-99.36% |
|  | Virus strains from immunosuppressed patients | 97.23%-98.51% | 98.09%-99.36% |
|  | Virus strains from immunocompetent patients | 98.30%-98.94% | 98.51%-99.36% |

MI45: A/Michigan/45/2015; BR02: A/Brisbane/02/2018
