# Supplementary material for: Phylogenetic analysis of HA and NA genes of influenza A viruses in immunosuppressed inpatients in Beijing during the 2018–2020 influenza seasons
Source: Virol J. 2023 May 26;20:101. doi: 10.1186/s12985-023-02067-2 (PMC10215044; doi:10.1186/s12985-023-02067-2)
Supplement: Supplementary file 3 — Additional file 3: Table 3. Nucleotide similarity of HA and NA genes of Acompared to vaccine strains. [file 12985_2023_2067_MOESM3_ESM.docx]

| **Supplementary Table 3** Nucleotide similarity of HA and NA genes of A(H3N2) compared to vaccine strains | | | |
| --- | --- | --- | --- |
|  |  | SI0019 | KA14 |
| HA | All virus strains | 96.71%-98.88% | 94.71%-99.76% |
|  | Virus strains from immunosuppressed patients | 96.71%-98.88% | 94.71%-96.94% |
|  | Virus strains from immunocompetent patients | 96.88%-98.88% | 96.41%-99.76% |
| NA | All virus strains | 95.67%-98.65% | 96.74%-99.50% |
|  | Virus strains from immunosuppressed patients | 95.67%-98.58% | 96.74%-99.50% |
|  | Virus strains from immunocompetent patients | 97.73%-98.65% | 98.51%-99.43% |

SI0019: A/Singapore/INFIMH-16-0019/2016; KA14：A/Kansas/14/2017
