# Supplementary material for: Phylogenetic analysis of HA and NA genes of influenza A viruses in immunosuppressed inpatients in Beijing during the 2018–2020 influenza seasons
Source: Virol J. 2023 May 26;20:101. doi: 10.1186/s12985-023-02067-2 (PMC10215044; doi:10.1186/s12985-023-02067-2)
Supplement: Supplementary file 4 — Additional file 4: Table 4. Amino acid similarity of HA and NA genes of Acompared to vaccine strains [file 12985_2023_2067_MOESM4_ESM.docx]

| **Supplementary Table 4** Amino acid similarity of HA and NA genes of A(H3N2) compared to vaccine strains | | | |
| --- | --- | --- | --- |
|  |  | SI0019 | KA14 |
| HA | All virus strains | 95.41%-98.24% | 93.83%-100.00% |
|  | Virus strains from immunosuppressed patients | 95.41%-98.24% | 93.83%-97.18% |
|  | Virus strains from immunocompetent patients | 97.00%-98.06% | 96.12%-100.00% |
| NA | All virus strains | 94.47%-98.09% | 96.17%-99.36% |
|  | Virus strains from immunosuppressed patients | 94.47%-98.09% | 96.17%-99.36% |
|  | Virus strains from immunocompetent patients | 97.02%-98.09% | 98.30%-99.36% |

SI0019: A/Singapore/INFIMH-16-0019/2016; KA14：A/Kansas/14/2017
